# Should free-text data in electronic medical records be shared for research? A citizens’ jury study in the UK

**DOI:** 10.1136/medethics-2019-105472

**Published:** 2020-05-26

**Authors:** Elizabeth Ford, Malcolm Oswald, Lamiece Hassan, Kyle Bozentko, Goran Nenadic, Jackie Cassell

**Affiliations:** 1 Department of Primary Care and Public Health, Brighton and Sussex Medical School, Brighton, UK; 2 Citizens’ Juries CIC, Manchester, UK; 3 Division of Informatics, Imaging and Data Sciences, School of Health Sciences, University of Manchester, Manchester, UK; 4 Jefferson Center, Saint Paul, Minnesota, USA; 5 Department of Computer Science, The University of Manchester, Manchester, United Kingdom

**Keywords:** stakeholder participation, medical text, natural language processing, text mining, privacy, healthcare

## Abstract

**Background:**

Use of routinely collected patient data for research and service planning is an explicit policy of the UK National Health Service and UK government. Much clinical information is recorded in free-text letters, reports and notes. These text data are generally lost to research, due to the increased privacy risk compared with structured data. We conducted a citizens’ jury which asked members of the public whether their medical free-text data should be shared for research for public benefit, to inform an ethical policy.

**Methods:**

Eighteen citizens took part over 3 days. Jurors heard a range of expert presentations as well as arguments for and against sharing free text, and then questioned presenters and deliberated together. They answered a questionnaire on whether and how free text should be shared for research, gave reasons for and against sharing and suggestions for alleviating their concerns.

**Results:**

Jurors were in favour of sharing medical data and agreed this would benefit health research, but were more cautious about sharing free-text than structured data. They preferred processing of free text where a computer extracted information at scale. Their concerns were lack of transparency in uses of data, and privacy risks. They suggested keeping patients informed about uses of their data, and giving clear pathways to opt out of data sharing.

**Conclusions:**

Informed citizens suggested a transparent culture of research for the public benefit, and continuous improvement of technology to protect patient privacy, to mitigate their concerns regarding privacy risks of using patient text data.

## Introduction

The use of medical data for secondary purposes such as health research, audit, care quality improvement and service planning is well established in the UK, and technological innovation in analytical methods for new discoveries using these data resources is developing quickly.^1–3^ Data scientists have developed, and are improving, many ways to extract and process information from medical records. This continues to lead to an exciting range of health-related discoveries, improving population health and saving lives across diverse areas such as improving health outcomes for people with learning difficulties, drug safety in children with chronic conditions and how to personalise treatments for cancer.^4^

Nevertheless, as the development of analytic technologies accelerates, this raises increasingly urgent questions about the suitability of the existing ethical and governance landscape.^5^ Inherent to ethics discussions is the need for greater understanding of public awareness, opinion and acceptance of this work, an area where the evidence often lags behind.^6 7^ One area where this is especially true is in the use of free text from medical records.

### What are the differences between structured and unstructured (free-text) data?

Much current health data research and innovation uses patient data stored in electronic health records (EHRs). EHR data can be structured or unstructured. Structured data are organised using a code set of predefined clinical concepts (eg, Read codes, SNOMED, etc) relevant to observations, diagnoses, treatments and other investigations or interventions. These types of data can be easily de-identified, formatted into databases and processed statistically at scale. This contrasts with unstructured medical data called ‘free text’, which is clinical information written in words, which are narrative in nature and not limited to predefined values or structures (table 1).

**Table 1:** Examples of structured and unstructured data within general practice medical records

Data item	Examples	
	Structured*	Unstructured
Time	dd/mm/yyyy 14 September 2017 Prescription start date: dd/mm/yy	‘Earlier today Mr X experienced chest pain’ ‘Operation scheduled on Tuesday’.
Symptoms	N242300 Neuropathic pain 1B1B.00 Cannot sleep—insomnia	‘…c/o shooting pain in upper right leg during the night, disturbing her sleep’
Diagnosis	C109912 Type 2 diabetes without complication	‘The patient has diabetes without complications’
Prescription	01040200 (BNF code for codeine phosphate 60 mg tablets)	‘Px codeine 60 mg PO qid×7 days’
Referral	8H4D.00 Referral to psychogeriatrician	Rev 4 w ?refer pyscho ger
Test	43F1.00 Rheumatoid factor positive	Rheumatoid factor was 42 IU/mL which is a positive result

*These codes represent Read codes, a UK-based, alphanumeric clinical coding system for general practice, and British National Formulary (BNF) codes, which represent the full list of medications available in the UK.

### Why is medical free text important for research?

Much influential epidemiological research has been published in the UK from general practice (GP) medical record databases, for example, on the safety of vaccines, of oral contraceptives and of medications taken during pregnancy.^8–11^ Typically, studies have relied on structured data, because GPs’ written notes and letters are not commonly available in these databases. However, this is problematic given that several studies have shown that clinical information is lost when medical data in the form of unstructured text are not used.^12–17^ Part of the problem is that clinical information is often missing from structured fields. This may be due to a variety of reasons including clinical uncertainty, stigma, loss of information between secondary and primary care, time pressures or poor clinician training in the coding structure. For example, in the case of UK general practice, many GPs describe choosing a ‘summary’ code, which is a keyword representing the main body of the consultation (table 1).^14^ The GP may then add text under the code to capture complexity, evolving circumstances, uncertainty and severity.^18^ If only the coded data are available, all of this additional clinical and contextual information may be lost to researchers who subsequently use GP patient data for research. In our previous research, we also found much clinical information, such as a new diagnosis, was incorporated into the record only within letters which came from specialist clinics in the hospital.^19^ However, these were often recorded under an administrative code such as ‘letter from specialist’ rather than a diagnostic code relating to the clinical information they communicated.^19^ It has been shown that, when using GP data, additional information about symptoms^20^ and date of diagnosis^19^ can be ascertained from text only.

Beyond general practice, certain types of patient data in the UK are generally only recorded as unstructured text. These include medical records produced within mental healthcare, where records are largely narrative in nature and very little is formally coded, as well as communications between doctors within and between hospitals, to and from primary care as well as between other hospital staff (eg, nursing handover notes). These data sources include imaging and pathology reports, discharge summaries and letters. Even where free text and coded data exist side by side, the addition of information from the text data to information which is coded can improve sensitivity of case definition in EHR research.^21^

### The current ethics and governance landscape for free text

While there has been real progress in developing text analytic technology in many domains, there are challenges to developing such technologies for medical or health research applications, due to the reluctance of patient data providers to give access to free text. Many text analysis research groups in the UK report that being refused access to medical text is the main barrier using these data to improve health and healthcare.^22^ The reluctance relates to concerns about patient privacy. Patient data are usually de-identified before being shared for research, following which they are usually held and analysed in a secure computing environment with only trusted individuals being allowed access. However, policy makers routinely judge that the risk of re-identifying patients from text data is too high for these data uses. While automated methods for stripping text of identifiers (of both patients and third parties) exist, they are not perfect, performing at 81%–99% sensitivity (recall) and 43%–99% precision,^23 24^ and consequently many data custodians refuse to share text outside of the clinical environment. In contrast, the few UK research groups that are situated within healthcare trusts and can access medical text which remains within the clinical environment, have established good track records in terms of technology development,^25^ protecting patient privacy^26^ and generating clinical insights.^27–29^

### What does the public think about their medical free text being used for research?

Two recent reviews examined studies of public views on sharing medical records for research.^6 7^ Both found that no previous study explicitly differentiated between text data and structured data within medical records. Aitken et al.^7^ found two studies in which participants indicated that they were more comfortable with the idea of their health data being turned into ‘*figures for doing stats*’^30^ and were uncomfortable with the idea of researchers ‘*having everything in there because it is not relevant*’.^30 31^ They also found a study on public views of using social media data for health research which mirrored these findings, in which participants indicated that if written posts were aggregated and used for statistics, participants were more likely to be agreeable to the research happening without their consent. Aitken 30 and Aitken *et al* 7 also reported that across studies, participants regarded certain types of data as more sensitive, including data relating to mental health, sexual health, sexuality and religion. However, no research has explored in detail with the public the specific question of whether they feel differently about their structured or unstructured patient data being shared for research. Addressing this gap in knowledge of public opinion is a crucial step towards informing and influencing an ethical approach to access to patient data in line with the evolving analytical techniques evident in this space.

### Why conduct deliberative research on this topic?

Deliberative research has been used widely when forging policy for health issues which may be controversial or for when there are complex ethical issues to resolve.^32^ Like many ethical issues, balancing privacy risks with the research benefits of sharing free-text data is a complex area with a lot of information and many arguments to consider. While surveys and focus groups provide useful information about what the public thinks on certain topics, they are less appropriate for gathering information on topics that the average member of the public might know little about. Public deliberation is an approach designed to capture in-depth and informed public perspectives on complex topics.^33^ Deliberative democracy in healthcare typically aims to achieve public engagement, shared decision-making and patient-centred care.^32^ Additionally, deliberative methods have previously been used to address issues of policy in health or medical research and data sharing.^34 35^

Research has already shown general public willingness for medical data to be shared for research for the common good.^6^ However, there is reason to believe that public may have different views on unstructured text data being analysed for research compared with structured data, as it has the capacity to tell more details of the patient story, provide additional contextual information and identify third parties (eg, family members).^30 31^ Thus, it may be harder to secure public approbation, also known as a social license, to use medical text data for research. According to social licence theory, organisations can engender trust from the public for schemes which may initially be controversial (such as data sharing), by voluntarily adhering to social codes of trustworthy and responsible behaviour that go beyond legal or regulatory frameworks and by honouring additional safeguards.^36^ Where the public are satisfied that the motivations of the organisation are trustworthy, they grant a ‘social licence’ to operate. It has been hypothesised that previous patient data-sharing initiatives, such as Care.data, failed precisely because they lacked a social licence for their operation.^37^

No evidence currently exists about what public perceptions are of medical free text being shared, processed and analysed. For example, it is plausible that additional privacy safeguards may be desirable when using text data compared with coded data. We therefore sought to gather detailed evidence on this topic for the first time. We chose a deliberative approach for this study, in order to inform participants about the complex issues, and to give them space to reflect on the topics and give their opinion. We planned and undertook a citizens’ jury asking a group of members of the public to deliberate on whether medical free text should be shared for research.

## Methods

### The citizens’ jury method

In a citizens’ jury, a broadly representative sample of citizens are selected to come together for a period of days, hear expert evidence, deliberate together and reach reasoned conclusions about questions posed to them. The method was developed by Ned Crosby, the founder of the Jefferson Center in the USA, in the 1970s.^38^

A citizens’ jury can tell policymakers what members of the public think once they have become more informed about a policy problem.^39^ Juries are a form of deliberative democracy, based on the idea that individuals from different backgrounds and with no special prior knowledge or expertise can come together and tackle a public policy question.^39–41^ Deliberative democracy is an egalitarian approach which encourages mutual recognition and respect and allows public negotiation of the common good.^39^ Citizens who take part are encouraged to deliberate in an environment free of ‘delusion, deception, power and strategy’, and in which all parties have an equal right to be heard.^42^ A citizens’ jury is a particularly relevant method for informing public bodies making value judgements. Juries are increasingly used for informing health policy on ethically complex topics such as genetic testing,^43^ use of patient data,^34^ screening services,^44^ case finding for stigmatised diseases^45^ and health service resource distribution, among others.^46^

Expert witnesses present information to the jury, and care is taken to make sure the full range of views is presented equivalently. The jury discussions are assisted throughout by impartial facilitators.

### Reducing bias in the jury

To make sure this jury was conducted appropriately and to reduce bias, the running of the jury was contracted to Citizens’ Juries CIC, a UK-based social enterprise who recruited the citizens and expert witnesses, and managed the project. Citizens’ Juries CIC worked in partnership with the Jefferson Center, who led the design and facilitation of the 3-day jury process. An external oversight committee was recruited, who reviewed all materials for the jury to identify potential bias, including presentations, questionnaires and planned activities. The oversight panel members were chosen for their knowledge of the topic and lack of conflict of interest in any particular jury outcome, and was made up of the NHS England Head of Data Sharing and Privacy, the Head of the Office of the National Data Guardian and the Director of Public Engagement for Connected Health Cities, an umbrella organisation which unities local health data and technology to improve health services in cities across the north of England.

The jury questions, and the consequent topics for witnesses and deliberations, were planned and refined over the course of 6 months by all authors. Prior to the jury, the panel reviewed the citizens’ jury questions and design, and much of the detailed jury documentation, including the jury questionnaires and the slides from the presentations by the expert witnesses, resulting in some changes to these materials.

### Participant recruitment

The jury was advertised on the Indeed job website (www.indeed.co.uk), on Brighton community base website (http://www.communitybase.org) and by word of mouth. Interested members were invited to apply to participate by filling in a questionnaire, which asked for information on gender, age range, ethnicity and educational attainment, as well as an Ipsos MORI survey question from a 2016 poll of the public commissioned by the Wellcome Trust^35^: “*How willing or unwilling would you be to allow your medical records to be used in a medical research study? The information given to researchers would not include your name, date of birth, address or any contact details*”.

In total, 227 members of the public responded to the advert by filling in the online survey. Candidates were chosen from this pool of applicants using a sampling logic of representativeness, and trying to achieve matches on gender, age ethnicity and educational attainment with UK census data for England.^47^ We also aimed to recruit a jury whose views on data sharing broadly reflected those in the wider population based on the Ipsos MORI question. Citizens’ jury members are often selected purely according to demographics, but occasionally on the basis of responses to questions on allied topics, where recruiters seek to bring a diverse range of voices within the jury.^46^ The stratified sample of recruited jurors closely reflected England’s demographic mix, and the range of views expressed in the Ipsos MORI survey. Healthcare professionals and specialists in law and information governance were excluded, as their specialist knowledge of the subject area may have resulted in other members of the jury deferring to them, and holding back from freely expressing their views.

Each juror was paid £300 for 3 days plus a £25 expense allowance. Three ‘reserve jurors’ were also recruited. One participated until lunch on day 1 and was paid £75. The two other reserves became full members of the jury, after one original juror called in sick and another left for personal reasons early on day 1.

### Expert witnesses

From the jury questions, the main information needs of the jury were identified, and an expert witness brief was developed (downloadable from http://healtex.org/jury/). Seven expert witnesses were chosen to provide relevant information about healthcare text analytics and research. The choice of witnesses was discussed extensively in the research team and the research team endeavoured to use local witnesses where possible, and to achieve diversity of demographics in the witnesses. Each witness answered questions posed by the jurors. They all presented slides which were reviewed for bias in advance by the oversight panel. Five expert witnesses were impartial witnesses who were instructed to present information to the jurors without trying to influence their views. These witness presentations covered:

1. Patient records: what they contain in codes and text, and how clinicians decide what to record, delivered by a clinician-researcher.
2. How free text can be de-identified by computers and how successful this is, delivered by a computer scientist.
3. Five processes by which information is extracted from medical free text to use for research, delivered by a health researcher.
4. Ethical issues around the use of patient data and free text specifically, delivered by a bioethicist.
5. The law around the use of patient data, delivered by a barrister also trained as a doctor.

In addition, a researcher made the case for the use of free text being shared for research, and a medical confidentiality campaigner made the case for being cautious about data sharing. We did not cover other data-sharing topics such as records linkage or commercial uses of patient data. Full documentation for the jury, including presentation slides, can be seen at http://healtex.org/jury/.

### The jury process

The 3-day jury programme was facilitated by a representative of the Jefferson Center, USA.

On the first day, patients gave informed consent for participation, and listened to an introductory presentation to explain the citizens’ jury method. They heard expert witness 1, and then undertook an exercise to anonymise a health record. They then heard expert witnesses 2 and 3, and a third witness (witness 5) engaged the jury in a session of question and answer with these two witnesses. The balancing witness was briefed to ask challenging questions to the experts on free text de-identification and information extraction, to attempt to counterbalance the potential bias that comes from explaining about uses and value of free-text data; as these witnesses may have been perceived as favouring the case for using free-text data.

On day 2, the jury deliberated further on the anonymisation and processing of free text, and then listened to presentations 4 and 5 (see above). In the afternoon, they heard the two witnesses who made the case for and against the sharing of free-text data.

On day 3, jurors worked together extensively in groups, and were encouraged to deliberate, listening and responding to the thoughts expressed by others. They deliberated on the jury questions, and prioritised their reasons for answering their questions in a certain way. They voted on the jury questions individually and anonymously. Jurors were not instructed on whether they should think about what is best for everyone or focus on what is best for themselves. They deliberated on how to generalise from the case example used in the questions, and worked together with facilitators to create a jury report. They filled in an end-of-jury questionnaire.

### Jury questionnaire

The questionnaire was developed specifically for this jury with the contribution of all authors over a 6-month period. The questions were tested with 10 members of the public in a half-day public workshop and feedback was received. There were five sections to the main jury questionnaire. See online supplementary appendix 1 for the jury questionnaire in full, and http://healtex.org/jury/ for full jury documentation.

10.1136/medethics-2019-105472.supp1 Supplementary data

#### Section 1: Tom scenario

The jury were presented with a case scenario about a man aged 43 years who registered newly with a GP practice after living abroad. Tom’s GP finds he has symptoms of diabetes and records information about this in the patient record. Later on, Tom has mental health symptoms and is seen in the local mental healthcare service. The local university wants access to both types of patient records for research purposes.

The jury were asked whether the GP practice and the mental health trust should release (a) the coded data about diabetes; (b) the free-text data about diabetes and (c) the free-text data about mental health. They were asked to give their reasons for any difference in response to these three questions. The response scale was: (a) ‘yes’, (b) only if Tom and the other patients can opt out, (c) only if Tom and the other patients can opt in, (d) no and (e) other.

#### Section 2: views on the method of de-identification of free text

In this section, the jury were asked three questions about how comfortable they felt about the de-identification of free text being undertaken by a person, a computer or a combination of a person and a computer. They responded on a 5-point Likert scale from comfortable to uncomfortable.

#### Section 3: views on methods of processing free text to extract information for research

In this section, the jury were asked five questions about processes for coding or analysing free text for research purposes. These included text being converted into structured data by a clinician or medical student, being used to train a computer algorithm to extract information from text or a computer algorithm extracting information from text unsupervised. It also included a scenario where researchers conducted a qualitative analysis on small bodies of medical text. Jurors responded to the scenarios on a 5-point Likert scale from strongly supportive to strongly unsupportive.

#### Section 4: summary question

Jurors were asked the question “You have heard reasons to support the process of anonymising, coding and using free-text data for health research, and reasons to be concerned about the process. Given these, to what degree do you support the use of free-text data from patients’ records for health research?” and responded on a 5-point Likert scale from strongly supportive to strongly unsupportive.

#### Section 5: reasons and suggestions

The jury were asked three questions to elicit qualitative data about reasons to support sharing of medical text, reasons to be concerned about sharing of medical text and suggestions about how these concerns could be overcome. Jurors deliberated on these three questions as a group and their responses to these questions were generated during a facilitated discussion and agreed as a group.

### End-of-jury questionnaire

At the end of the jury questionnaire, the participants were asked the Ipsos MORI screening question once more, as well as four further questions: how interesting they found the jury, whether facilitators tried to influence them to particular conclusions, whether any other speakers (witnesses) tried to influence them to particular conclusions and whether the information they were given was a fair balance of information.

### Data analysis

Because only 18 participants took part, quantitative data analysis was restricted to descriptive statistics, including simple counts and percentages. All reasoning given in the final jurors’ report has also been presented verbatim to illustrate the jurors’ responses. Limited qualitative data were collected during deliberations of the jury, and so no formal analysis of the deliberations was carried out (as is sometimes provided in other juries carried out for research purposes).

## Results

### Participant characteristics

**Figure 1:**
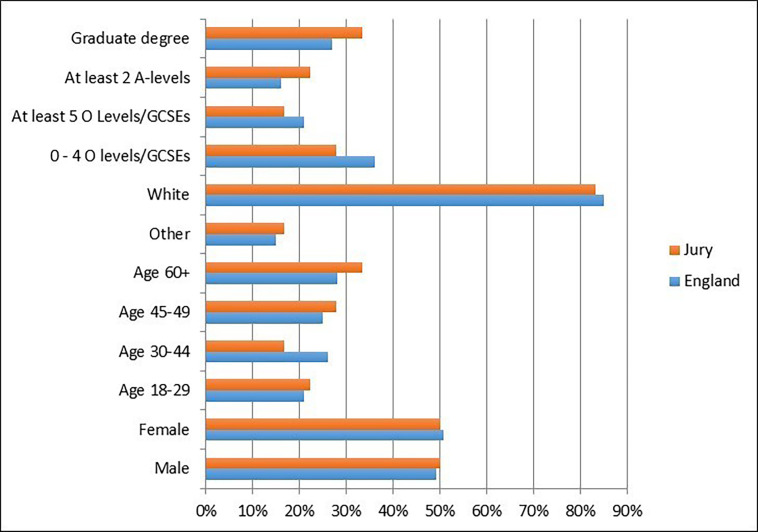
Demographic make-up of jury against average for England (UK Census 2011).

Eighteen people were recruited from around Brighton, UK, a city of around 290 000 inhabitants on the southern coast of the UK. Of the 18 jurors, 11 people responded to an advertisement placed on the Indeed jobs website, 5 to an advert on Brighton’s Community Base website and 1 applied through word of mouth. Five people were in full-time or part-time employment, six were self-employed, three were unemployed, two were retired and two self-classified as having an ‘other’ employment status. The sample chosen was controlled for gender, age range, ethnicity (in terms of white/other) and educational attainment, and matched closely the demographics of people in England (as recorded in the UK Census 2011) and the range of views expressed in the Ipsos MORI survey. Figure 1 shows the demographics of the 18 people who completed the 3-day process.

### Willingness of participants to share health data in general

Participants were asked to respond to the following Ipsos MORI survey question as part of the application to the jury and after the jury^35^: *“How willing or unwilling would you be to allow your medical records to be used in a medical research study? The information given to researchers would not include your name, date of birth, address or any contact details”.* Responses are shown in table 2.

**Table 2:** Summary of juror answers

How willing or unwilling?	Recruitment questionnaire	End-of-jury questionnaire
Very willing	9	9
Fairly willing	6	9
Do not know	0	0
Fairly unwilling	2	0
Very unwilling	1	0

The figures mentioned in table 2 suggest a general movement towards greater willingness to allow use of medical records for research. However, over the 3 days, 12 out of 18 people changed their minds and gave a different answer to the question to the one they originally provided during jury recruitment, with 7 people becoming more willing and 5 becoming less willing.

### Willingness of participants to share free-text data

In response to the summary question at the end-of-jury process, “to what degree do you support the use of free-text data from patients’ records for health research?” 6 jurors responded ‘strongly supportive’ and 12 jurors responded ‘fairly supportive’. No jurors gave neutral or unsupportive responses. Reasons given by the jurors, assumed to underpin these responses, are shown in box 1, although these reasons to support, or be concerned about, text data sharing were generated after responding to the jury questions.

Box 1 **Reasons and suggestions**

**The main reasons to support the process of anonymising, coding and using free-text data for health research:**

- There is a large amount of free-text data in patient records, particularly for mental health cases. This free text can be richer than coded data, adding ‘flesh’ to the coded data within a patient record.
- This richer data can enable better research that could lead to better treatments, improve care and may save lives.
- There is a low risk of re-identification when processing free text if proper procedures are followed.
- An opt-out system gives a larger, more representative sample of the population for research than an opt-in system which can lead to more accurate research and better results.
- When millions of records need to be processed by computer and there may be too many for humans to process effectively, these processes can support better research.

**The main concerns about the process of anonymising, coding and using free-text data for health research:**

- If people believe their data are unsafe, they may withhold important information when seeing their doctor.
- The law requires ‘fair processing’—patients must be informed of the uses of their data but sometimes they are not.
- There is a lack of awareness about how patient data are used, or by whom and that patients can opt out.
- People who might otherwise be willing to share information may be less willing to do so if they are unable to either give permission or be informed and able to opt out.
- Data processed to remove identifiers does not always mean it is completely anonymous.
- Free-text data are sensitive and inherently more identifying than coded data.
- Computer programs are currently unable to remove identifiers to an acceptable level with 100% accuracy.
- Free-text patient data could contain information about other patients, judgements, offhand comments and other data requiring interpretation, and could be misinterpreted by researchers.
- There is a procedure in place (section 251) to ask for legal approval to process free-text data without requiring consent in specific scenarios.
- Despite safeguards that might be in place, IT and data protection systems may be at risk of being accessed by third parties who seek unauthorised access to records and data.

**Suggestions for how these concerns could be overcome:**

*Transparency:* patients should be comprehensively informed at the outset about how, when and under what conditions their free text might be processed, anonymised, coded and analysed for research purposes. This should include:

- Information that communicates their rights (to file complaints, to access their own information, etc).
- A privacy statement.
- How data will be protected from breaches during processing, analysis and once research is completed.
- Whether or not and how their information will be anonymised.
- Who would access it and for what purpose; and plans for long-term storage or management of their data.

Researchers should communicate how decisions are made about who, why and under what circumstances patients’ data and records are being used in language that is accessible and easy to understand.

Efforts could be undertaken to involve patients in various elements of research and ethics decision-making (such as patients sitting on ethics boards) so that these processes are more open and transparent.

Providing an option for people to access (published) research which uses their records or data might be useful in maintaining trust.

*Technology:* there has to be continuous improvement in the methods used for coding, anonymising and processing free text, as well as in systems for safeguarding IT systems that secure access to data to improve performance, data protection and public confidence.

### Responses to jury questionnaire

All participants responded individually to the questions relating to the case scenario about Tom, who had diabetes, as well as depressive symptoms and auditory hallucinations, and was seen at the GP clinic and the mental health trust. Their aggregated responses are shown in table 3. Some comments were also made on questionnaires about the need for opt-outs to be made easy and transparent, and the need to fully inform patients, in relation to (1A), (2B) and (3B). However, it should be noted that some members of the jury may not have fully understood the difficulties of achieving easy and fully informed opt-out.

**Table 3:** Responses to data-sharing questions from Tom scenario (see online supplementary appendix 1 for full questionnaire)

Question	Participants’ responses			
	(A) Yes	(B) Only if Tom and the other patients can opt out	(C) Only if Tom and the other patients can opt in	(D) No
(1) Should Anytown health centre agree to release the coded data items about Tom and all the other patients in the practice with suspected or confirmed type 2 diabetes?	8	8	2	0
(2) Should Anytown health centre also agree to release the free-text data items about Tom and all the other patients in the practice with suspected or confirmed type 2 diabetes?	4	12	2	0
(3) Should Anytown mental health trust agree to release the free-text data items about Tom and all the other patients in the trust who hear voices or have hallucinations?	4	12	2	0

Reasons for differences in response between question 1 and question 2 were given by four participants. These are all shown in box 2. Nobody indicated that they responded differently to the free-text sharing question about diabetes (question 2) compared with the free-text sharing question about mental health (question 3).

Box 2 **Reasons given for different response to free-text question**

- ‘There may be more sensitive information including about other people in free text and some identifiers may slip through’.
- ‘Because in the first case it is coded data but free text in the second’.
- ‘Further information to Tom as to how data will be used that is, more people with diabetes will gain better healthcare or a cure may be found’.
- ‘Because free-text data are far more sensitive than coded information is and can be easily readable and leaked more easily’.

### Views on human or computer de-identification of free text

The jury heard about the processes used for de-identifying free-text data for research. Participants indicated they were largely comfortable with the idea of de-identification being conducted by a human or a computer but overall the combination of human and computer was most preferred (table 4). The particular concerns they raised on the topic of de-identification were that data processed to remove identifiers does not always mean it is completely anonymous; free-text data are sensitive and inherently more identifying than coded data; computer programs are currently unable to remove identifiers to an acceptable level with 100% accuracy and that free-text patient data could contain information about other patients, judgements, offhand comments and other data requiring interpretation, and could be misinterpreted by researchers (box 1).

**Table 4:** Views on de-identification of free-text data

*How comfortable are you with de-identification of free-text patient data:*	Participants’ response				
	Comfortable	Somewhat comfortable	Neither	Somewhat uncomfortable	Uncomfortable
I. Where done by a person (researcher or healthcare professional)?	5	11	0	2	0
II. Where done by a computer?	5	12	0	1	0
III. Where done by a combination of a person and a computer?	8	10	0	0	0

### Views on different methods of processing free text

Participants of the jury were largely supportive of the different methods of processing free text for research with which they were presented (table 5). Jurors were most supportive of the cases where a computer algorithm was trained to extract the information needed for research.

**Table 5:** Views on methods of processing free text

*How supportive are you of each of the processes for extracting clinical information from free text, as described below?*	Participants’ response				
	Strongly supportive	Fairly supportive	Neither	Fairly unsupportive	Strongly unsupportive
I. Where text is coded by the healthcare professional (eg, GP or nurse) who provides care and records the free text?	7	8	1	2	0
II. Where text is first anonymised by computer and/or person, then provided to a research team who will read the free text in order to gain a deep understanding of a specific thing (qualitative analysis)?	7	10	0	1	0
III. Where text is first anonymised by computer and/or person, then coded by a medical student and checked by a healthcare professional from the research team?	11	6	0	1	0
IV. Where text is first anonymised by computer and/or person, then coded by a medical student and checked by a healthcare professional, and then used to develop a computer program which would automatically code other patient records for research?	10	8	0	0	0
V. Where text is first anonymised by computer and/or person, then automatically coded by a computer program and checked by a healthcare professional?	7	10	1	0	0

### Reasons for and against sharing free text for research

Participants deliberated as a group and proposed the following reasons to support or be concerned about the use of medical free-text data for research (Box 1). They also proposed ways of addressing these concerns. All jury members read and agreed the final list of reasons and suggestions before they were added to the final jury report.

### Perceptions of bias in the jury process

At the end-of-jury questionnaire, participants were able to indicate their perception of how biased the jury process had been towards one verdict over another. These results are shown in table 6. It was particularly important that the facilitators did not bias the jury. In comparison, two of the witnesses, putting the case for and against, were specifically tasked with trying to convince the jury in their favour. No participants perceived that the facilitators tried to influence the juror conclusions, whereas six jurors felt that other presenters had ‘perhaps occasionally’ tried to influence them. Fifteen jurors felt the information was a fair balance and three felt that there was some bias in favour of sharing free text.

**Table 6:** Perception of bias in the jury

Question	Participants’ response				
	Not at all	Perhaps occasionally	Sometimes	Often	Very often
Did you ever feel that the jury facilitators tried to influence you towards particular conclusions?	18	0	0	0	0
Did you ever feel that anyone else (other than the other members of the jury) tried to influence you towards particular conclusions?	12	6	0	0	0
	Yes, overall it seemed a fair balance.	No, overall I thought there was some bias in favour of using free-text data for research.	No, overall I thought there was some bias in favour of protecting information		
Do you feel that overall that the information you were given provided a fair balance of information about using free-text data for research and protecting information?	15	3	0		

## Discussion

### Summary of findings

To the authors’ knowledge, this is the first in-depth enquiry specifically investigating how the UK public feel about their medical free text being shared outside the National Health Service (NHS) for research, and results can be used to inform national policy. The 18 citizens who participated were largely in favour of these data being shared for research using an opt-out model, although they did raise some concerns about patient privacy and gave some suggestions to mitigate these concerns.

Importantly, these results suggest that when fully informed, the public have similar views about their free text and coded data being shared for research. Jurors’ views were similar to those found by other citizens’ juries on sharing health data in general. For example, a UK study in which two juries were held in Manchester in 2016,^34^ to investigate public opinion towards the use of health data for research, found that 33 out of 34 were in favour of this, with the majority favouring an opt-out process. Other forms of qualitative research, such as Clerkin *et al*,^48^ have found that participants are overall positively inclined towards their patient records being used in research for the ‘greater good’, although participants expressed concerns about personal information being ‘leaked’. Participants in the focus groups in the study by Spencer *et al* 49 were supportive of sharing their anonymised electronic patient record for research, and likewise, participants in the focus group in the study by Hill *et al* 50 became more accepting of data sharing after being given information on selection bias and research processes. This suggests that a deliberative process, where participants are informed, does have the capacity to change participants’ views, and that the majority (though not all) become more positive about data sharing.

In our study, while showing general willingness to share, participants had several caveats about the use of medical free text for research. The key concerns were that patients are currently unlikely to be aware of the uses of their data beyond their immediate care because data uses are not transparent; that unauthorised third parties may be able to gain access to the data and that if patients feel like their medical confidentiality is being breached they are less likely to disclose important information to their doctor. These concerns largely map on to those found in other studies, for example, in the Manchester juries,^34^ participants felt that without a clear understanding of who would be regulating the data and making decisions about access, it would be difficult to support the sharing of data for research, and participants were concerned that organisations without proper permission or legal authority may access the data.

As our study was the first to ask participants specifically about sharing medical text, it is the first to draw out and compare key concerns relating to text as opposed to structured data. Despite appreciating that free text could not be perfectly de-identified, the majority of jurors believed the benefits of sharing data for research outweighed the privacy risks. Jurors did explicitly state the need for a culture of continuous improvement in de-identification methods, which appears to be a novel suggestion not previously offered by other juries. The jury members were able to understand the distinction between text and structured data and specifically evaluate the associated benefits and privacy risks. They noted that free text could contain richer, or more accurate information which may enable better research that could lead to better treatments, improve care and save lives. However, they acknowledged that it could not be perfectly de-identified and that the risk to privacy by sharing text data was greater than for sharing coded data. Of particular interest, the jury recognised that free-text patient data could contain information that could be misinterpreted by researchers and felt that its use should be approached with caution.

### Implications for health research and data policy

This informed group of citizens largely agreed that, with appropriate safeguards in place such as computerised de-identification and access restricted to appropriate persons, the benefits of sharing medical text from patient records outweighed the risks to individual privacy. Medical free text is not usually shared outside the NHS clinical care environment at the current time, despite growing research capabilities to extract information from it. These findings give the first evidence to policy makers about public opinion, which can be taken into account for making policy decisions about text sharing. Importantly, we provide evidence to policy makers that a well-informed public is likely to support sharing medical text data for research for the public good, with appropriate safeguards. These findings challenge existing data-sharing practices. However, the jury participants asked to give ‘suggestions’ for safeguards to mitigate their concerns, rather than ‘conditions’ within which they would be willing to share. We cannot know, therefore, if their suggestions are conditions for implementation of data-sharing, or simply articulated guidance for a best-case scenario.

In the light of these findings, and the fact that data regulations do not distinguish between free-text and structured data, it is interesting and arguably surprising that data providers are almost uniformly unwilling to allow researchers access to free text. Data providers are an informed group of decision makers who one might expect to have similar views to a citizens’ jury. However, free text is usually routinely redacted from datasets because it is seen as an unknown that might contain identifying data, with evidence that decisions are becoming more cautious in recent years (eg, the UK-based Clinical Practice Research Datalink (CPRD) used to provide free text to researchers but stopped this service in 2016; text is no longer held by CPRD or available to researchers^51^). This suggests that their motive for extreme caution, beyond what is practised for structured data, may be motivated by an anxiety about public perceptions, in the absence of evidence on likely public views, and by a currently unquantifiable risk to patients’ privacy of de-identified text data, in a climate where any kind of leak or breach could lead to loss of public trust and high financial penalty.

When and if national policy on sharing medical text is updated and reconsidered, it should respect the suggested mitigations proposed by this jury, which are well reasoned on the evidence that was presented to them. These mitigations include provision of patient-facing information to ensure complete transparency of data usage, such as posters clearly displayed in patient areas, or patient information leaflets provided on registration. This should explain a simple pathway for patients to opt out of having their de-identified free text shared. There should also be well-defined routes for patients who wish to get involved in decision-making bodies such as data providers and regulators, and information about patient representatives acting on behalf of patients as a group. Interestingly, the jury participants suggested healthcare text researchers should develop and make transparent a ‘culture of continuous improvement’ in technology for de-identification and information extraction of medical text, given that methods for de-identification and information extraction currently operate with certain levels of inaccuracy. These recommendations tie in with the theory of social licence, whereby honouring additional safeguards such as these, over and above any legal requirements, may help to engender trust and maintain transparency and secure societal approval for the research.

### Future research directions

This is the first step in understanding how the public views the sharing of their medical text for research. Although the participants in our study became well informed, nonetheless we are only able to show the view of 18 members of the public. We would need to scale up this research in the future to a wider pool of participants to be sure that these findings are not limited to particular attributes of the current jury.

Additionally, despite thorough communication throughout the jury of the majority of information needed to base their decisions on, presenters were not always able to answer some of the jury’s questions. For example, it is not currently known how likely various risks to privacy are, such as the chance of re-identification from free text, because so little data have previously been shared, thus there is no open-source data on breaches as a result of data sharing. In the future, to reassure patients, it would be good for the text analysis community to develop estimates of the likelihood of a patient being re-identified in various circumstances.

Public views are complex, and interpreting them to guide policy can be difficult. Some of us have previously argued that a concise ethical framework such as Beauchamp and Childress four principles,^52^ can act as a focusing lens, and that the public’s attitude to health data sharing can be interpreted through this lens.^6^ Respect for autonomy of individuals can be enacted through a meaningful and transparent opt-out policy, in line with national strategies for other sources of health data. Principles of justice should be written into any data-sharing agreements drawn up with research or commercial organisations outside the health service. Evidence that using free-text data for health research will result in the public good (beneficence) is currently sparse, and it is therefore imperative for the research community to bring together a body of evidence showing the potential benefits to patients of the use of their free-text data in research. The possibility of individual or collective harm (maleficence) should also be studied; potential harms from data sharing have been articulated by the public as identity theft, discrimination in employment, pension eligibility or insurance, stigmatising judgements in clinical settings or the community and the use of EHR data for financial profit-making.^6^ The route to these harms is usually posited to be through re-identification of the individual from their de-identified data. It is not clear how easily patients can be re-identified from imperfectly de-identified text data which is nevertheless stored in an NHS or university data safe-haven, nor who would have the motivation to do this. Quantifying the risk of re-identification by a series of tests, perhaps on simulated patient documents, might be one way in which we can better inform patients, data custodians and regulators about the true nature of privacy risks; this has been attempted on structured datasets.^53^ Increasing trustworthiness of research endeavours with patient data through transparency, and inclusivity of all stakeholders in the research process, remains paramount to ethical practice in this field.^54^

### Strengths and limitations

One of the strengths of this study is its use of a recognised deliberative method which has been replicated many times. In addition, the original developers of this method were involved in the design and delivery of this jury, in order to make sure all aspects of the technique were followed appropriately.

A criticism of citizens’ juries is the risk of bias being introduced when the presentations are given. We included an independent oversight committee in the development of materials to reduce (but not eliminate) bias, and this committee did request changes to several of the presenters’ materials. Despite this, some bias was perceived by participants. It may be difficult to avoid a perception of bias from the impartial witnesses, as we necessarily chose experts working in the field of text analytics, who are therefore more likely to be positive about the use of medical data for this purpose.

In addition, we asked members of the public to become skilled in evaluating and deliberating about difficult and complex material in <3 days. This was a jury full of complexity and nuance, so it is possible that presentations missed out some important points that would have influenced decision-making, or there may have been misunderstandings among jury members. We tried to reduce this chance as much as possible by allowing every witness to be questioned by the jurors and by other witnesses in some cases. In addition, participants were allowed to question one witness after the jury had a day to discuss her presentation among themselves. Thus, we believe most jurors were given enough information to make an informed decision.

One potential criticism of our method of evaluating perception of bias at the end of the jury could be the wording of the question “Did you ever feel that anyone else (other than the other members of the jury) tried to influence you towards particular conclusions?”; this could have been worded explicitly to exclude the witnesses who made persuasive cases for and against the use of free-text data. The very positive responses to this, suggesting a lack of perceived bias, may suggest jurors were subject to social desirability bias in their response to these particular questions.

Further limitations are the very limited collection of qualitative data such as audio-recordings of the deliberations. This limited our ability to make sense of the jury’s conclusions, and also to understand the thought process which brought the jury members to their final decisions. We chose to compose our sample with the same distribution of views on data sharing as was found in the Ipsos MORI survey.^35^ It is possible that using this data-sharing question as part of the selection process could have introduced bias, as the population majority view is largely in favour of data sharing. Conversely, there remains the possibility that views on data sharing in general would not directly predict the jury’s views on free-text data specifically, as we expected the jury members to have qualitatively different views on this format of data.

Finally, one of the limitations of this jury is that it did not address other related questions about health data sharing such as the use of medical text data by private or profit-making companies, the linkage of one set of patient records with another source of clinical or administrative data or sharing patient generated free text from social media. All the presentations were focused on public sector researchers such as those at universities, and on healthcare or clinic generated text. Thus, we cannot say how the public would feel about sharing different data with other types of research groups.

## Conclusions

An informed group of 18 citizens were largely in favour of sharing of medical free text research if patients were given full information and had the chance to opt out. They acknowledged the slight risk to patient privacy when medical text data were shared, and suggested that a transparent culture of research for the public benefit, and commitment to continuous improvement of technology to protect patient privacy, would assuage their current concerns about free-text data sharing. These rich findings can be taken forward to inform UK national policy around sharing NHS patient data for research.
