# Supplementary material for: Should free-text data in electronic medical records be shared for research? A citizens’ jury study in the UK
Source: J Med Ethics. 2020 May 26;46(6):367–77. doi: 10.1136/medethics-2019-105472 (PMC7279205; doi:10.1136/medethics-2019-105472)
Supplement: Supplementary data [file medethics-2019-105472supp001.pdf]

Introduction

Please read the following scenario about Tom and his health record. You will be shown the contents of his health record as it builds up over the course of the story. Your job is to decide what data from Tom's record should be used for health research, and what Tom should be told and/or asked when data from records about Tom are used.

Tom registers with a GP

Tom and his family emigrated from the UK soon after Tom was born. Aged 43, Tom returns to England, finds a job and settles in Anytown. Soon after arriving, Tom registers with a GP. He has no health records from the previous countries he has lived. He registers with a large local practice, Anytown Health Centre. He provides some basic details to the GP receptionist, including his full name, date of birth, and his new address. The receptionist suggests that Tom makes an appointment to see Dr. Jones, explaining that he can provide Dr. Jones with his medical history.

Tom sees the GP

At his appointment, Tom tells Dr. Jones what he knows about his medical history. He is not aware of his parents and other close family suffering from particular illnesses, except that his mother had type 2 diabetes. Tom explains he is overweight, and that he gets thirsty, and urinates often. Dr. Jones pricks his finger, and tests Tom's blood with a special strip. Dr. Jones notes down these symptoms, the high blood glucose level result, and the suspected diagnosis of type 2 diabetes. She asks Tom to attend the next diabetes clinic at the practice, and to avoid sugary foods in the meantime.

A diabetes research team approaches the practice asking for data

Anytown Health Centre takes an active interest in health research, and has close ties with Anytown University's Department of General Practice. The university has a signed data sharing agreement with the practice that has been approved by Anytown University's research ethics committee. The agreement states the measures the university will take to protect the data properly, and states that any proposed new use of the data must be approved in advance by Anytown Health Centre and the university's research ethics committee.

Sue Stark, Anytown Health Centre's manager, receives a letter from Prof. Smith, the lead researcher for a university project approved by the research ethics committee. Prof Smith wants to use anonymised general practice data meaning the risk of identifying a patient is very low. This is achieved by removing patient identifiers and restricting data access to the research team. Prof. Smith will use the anonymised data to identify characteristics of patients with both suspected and confirmed type 2 diabetes. This could enable the researchers to identify early signs of the disease, helping GPs and patients to spot type 2 diabetes earlier, and reduce complications.

Sue emails Prof. Smith to say she will check this with the GPs. She asks the researcher to send a list of the items he is seeking. Prof. Smith replies saying he wants data about all

patients with suspected and confirmed type 2 diabetes. Some of the data items are recorded by GPs as codes (e.g. “1234” for type 2 diabetes), and some in free text boxes (e.g. “gets very thirsty at night”). The codes for all of the patients’ other diagnoses are also required. The GPs review the list of data items and agree that there would only be a very small risk of the researchers discovering the identity of a patient from the coded data items.

Questions about the Tom scenario:

Q1 Should Anytown Health Centre agree to release the coded data items about Tom and all the other patients in the practice with suspected or confirmed type 2 diabetes?

a) Yes
b) Only if Tom and the other patients can opt out
c) Only if Tom and the other patients can opt in
d) No
e) Other (please specify)

If you chose “other”, please explain. [50 words maximum]

The GPs are less sure about the free-text data because they do not know what sensitive or revealing information it might contain. Sue goes back to check this with Prof. Smith. He says that the university has software that automatically removes text that could be used to identify a person. Prof. Smith also says that the data counts as anonymised in law as the risk of identifying a patient is very small.

Q2. Should Anytown Health Centre also agree to release the free-text data items about Tom and all the other patients in the practice with suspected or confirmed type 2 diabetes?

a) Yes
b) Only if Tom and the other patients can opt out
c) Only if Tom and the other patients can opt in
d) No
e) Other (please specify)

If you chose “other”, please explain. [50 words maximum]

Q3. If your answer to Q2 is different to your answer to Q1, please give reasons for your answers. [50 words maximum]

Tom hears voices

When speaking to the nurse at the diabetes clinic, Tom mentions that he is feeling low. The nurse recommends he discusses this with his GP, and so Tom makes an appointment with Dr. Jones. At the appointment, Tom tells Dr Jones that he has been feeling low but also that he has been hearing voices: imagining he is having conversations with people he once knew. Dr. Jones suggests that it would be useful for Tom to talk to a specialist mental health practitioner

at Anytown Mental Health Trust. Dr. Jones refers Tom to the mental health trust to have an assessment and discuss what might be done to address his symptoms.

Some time later Tom is assessed by Ahmed Hussein, a psychiatric nurse at the Anytown Mental Health Trust. They talk about Tom hearing voices. Ahmed begins to build a picture of when this happens and how it affects Tom's life, making notes in Tom's record. Tom says the imaginary conversations are not usually distressing, and that the main problem is that they interfere with his concentration. He is particularly concerned not to lose his new job. Ahmed explains that medication and cognitive-based therapy are options that may help him control the voices. They agree that Ahmed will arrange for Tom to see a psychiatrist within the mental health trust. After Tom has left, Ahmed types up a summary of what Tom has told him, saves it within a new record for Tom within the mental health trust's patient record system. Ahmed also creates a referral letter (explaining that Tom hears voices) for Tom to be seen by the psychiatrist.

Researchers request data to investigate how hallucinations affect daily life

Prof. Brown, one of Prof. Smith's colleagues at Anytown University, is doing a research study about how hearing voices and having hallucinations affects people's lives. She has been looking at messages on internet forums for people who have times when they hear voices and have hallucinations. Some people posting online say that these episodes interfere with their work, and that this is often overlooked, or not addressed within the care decisions made by healthcare professionals.

Prof Brown needs some basic data about patients who have experienced hallucinations and heard voices, like age and gender, and some coded data including all their previous medical diagnoses. She also wants the free-text notes that are recorded on the mental health trust patient record system. This is because the coded data does not have all the details needed for her research. The free-text will be analysed by computer software to strip out identifying information such as names and dates of birth. The usual protections in place at Anytown University will also apply. Only the research team will be given access to this sensitive data. Prof. Brown suggests that with all the protections in place, there is only a very small chance of identifying a patient from the data, so the data set she requires counts as anonymised in law.

All this is explained in a letter to the research department at Anytown Mental Health Trust. The research lead in the trust brings it to the next senior management meeting where the issue is discussed thoroughly.

Q4. Should Anytown Mental Health Trust agree to release the free-text data items about Tom and all the other patients in the trust who hear voices or have hallucinations?

a) Yes
b) Only if Tom and the other patients can opt out
c) Only if Tom and the other patients can opt in
d) No
e) Other (please specify)

Q5. If your answer to Q4 is different to your answer to Q2, please give reasons for your answers. [50 words maximum]

Now some general questions about the use of free-text data:

Q6. How comfortable are you with anonymisation of free-text patient data:

I. Where done by a person (researcher or healthcare professional)?
   a. Comfortable
   b. Somewhat comfortable
   c. Neither comfortable nor uncomfortable
   d. Somewhat comfortable
   e. Uncomfortable

II. Where done by a computer?
   a. Comfortable
   b. Somewhat comfortable
   c. Neither comfortable nor uncomfortable
   d. Somewhat uncomfortable
   e. Uncomfortable

III. Where done by a combination of a person and a computer?
   a. Comfortable
   b. Somewhat Comfortable
   c. Neither comfortable nor uncomfortable
   d. Somewhat uncomfortable
   e. Uncomfortable

Q7 You have heard reasons to support the process of anonymising, coding and using free-text data for health research, and reasons to be concerned about the process. Given these, to what degree do you support the use of free-text data from patients' records for health research?

a. Strongly supportive
b. Fairly supportive
c. Neither supportive not unsupportive
d. Fairly unsupportive
e. Strongly unsupportive

Q8 You have heard about several different ways in which free-text data can be anonymised, coded and used for health research. How supportive are you of each of these processes?

I. Where it is coded by the healthcare professional (e.g. GP or nurse) who provides care and records the free-text
   a. Strongly supportive
   b. Fairly supportive

   c. Neither supportive not unsupportive
   d. Fairly unsupportive
   e. Strongly unsupportive

II. Where it is first anonymised by computer and/or person, then provided to a research team who will read the free text in order to gain a deep understanding of a specific thing (qualitative analysis)
   a. Strongly supportive
   b. Fairly supportive
   c. Neither supportive not unsupportive
   d. Fairly unsupportive
   e. Strongly unsupportive

III. Where it is first anonymised by computer and/or person, then coded by a medical student and checked by a healthcare professional from the research team
   a. Strongly supportive
   b. Fairly supportive
   c. Neither supportive not unsupportive
   d. Fairly unsupportive
   e. Strongly unsupportive

IV. Where it is first anonymised by computer and/or person, then coded by a medical student and checked by a healthcare professional, and then used to develop a computer program which would automatically code other patient records for research
   a. Strongly supportive
   b. Fairly supportive
   c. Neither supportive not unsupportive
   d. Fairly unsupportive
   e. Strongly unsupportive

V. Where it is first anonymised by computer and/or person, then automatically coded by a computer program and checked by a healthcare professional,
   a. Strongly supportive
   b. Fairly supportive
   c. Neither supportive not unsupportive
   d. Fairly unsupportive
   e. Strongly unsupportive
   f.

Note that in all the cases above apart from II., codes are created from free-text data and used for research.

Q9. What are the main reasons to support the process of anonymising, coding and using free-text data for health research?

(100 words maximum)

Q10. What are the main concerns about the process of anonymising, coding and using free-text data for health research?

(100 words maximum)

Q11. Can you suggest how these concerns could be overcome?

(100 words maximum)
